# Expression of dengue virus and Zika virus NS2B-NS3pro constructs alter cellular fatty acids, but co-expression with a Zika virus virus-like particle is detrimental to virus-like particle expression

**DOI:** 10.1186/s13104-023-06572-z

**Published:** 2023-10-27

**Authors:** Suwipa Ramphan, Nathamon Yimpring, Chontida Tangsongcharoen, Suthatta Sornprasert, Atitaya Hitakarun, Wannapa Sornjai, Sittiruk Roytrakul, Atikorn Panya, Duncan R. Smith

**Affiliations:** 1 Institute of Molecular Biosciences, Mahidol University, Salaya, 73170 Thailand; 2 Department of Medical Technology, Faculty of Allied Health Sciences, Burapha University, Chonburi, 20130 Thailand; 3 Functional Proteomics Technology, National Center for Genetic Engineering and Biotechnology (BIOTECH), Thailand Science Park, Pathumthani, 12120 Thailand; 4 Food Biotechnology Research Team, Functional Ingredients and Food Innovation Research Group, National Center for Genetic Engineering and Biotechnology (BIOTEC), Science Park, Pathumthani, 12120 Thailand

**Keywords:** Dengue virus, Zika virus, NS3 protease, Lipid, Fatty acid methyl esters, Virus-like particles

## Abstract

**Objective:**

Studies have shown that *Flavivirus* infection remodels the host cell to favour viral replication. In particular, the host cell lipid profile is altered, and it has been proposed that this process alters membrane fluidity to allow wrapping of the outer structural proteins around the viral nucleocapsid. We investigated whether expression of the Zika virus (ZIKV) and dengue virus (DENV) protease induced alterations in the cellular lipid profile, and subsequently whether co-expression of these proteases with VLP constructs was able to improve VLP yield.

**Results:**

Our results showed that both ZIKV and DENV proteases induced alterations in the lipid profile, but that both active and inactive proteases induced many of the same changes. Neither co-transfection of protease and VLP constructs nor bicistronic vectors allowing expression of both protease and VLP separated by a cell cleavable linker improved VLP yield, and indeed many of the constructs showed significantly reduced VLP production. Further work in developing improved VLP expression platforms is required.

**Supplementary Information:**

The online version contains supplementary material available at 10.1186/s13104-023-06572-z.

## Introduction

Virus-like particles (VLPs) offer an alternative to genetic material based vaccines and a number of commercial VLPs are available to protect against hepatitis B virus, (e.g. Engerix-B®) human papillomavirus (e.g. Gardasil®) and hepatitis E virus (e.g. Hecolin®). We have previously shown that production of dengue vius (DENV) VLPs is markedly less efficient than the production of virions by natural infection [1]. It is well established that flavivirus infection results in remodeling of the host cell to promote viral replication, and this includes remodeling of membranes and alteration of lipid metabolism [2]. It is believed that alterations of the lipid composition of the cell is necessary to increase membrane fluidity to allow membranes to bend sufficiently to allow formation of the viral particle [3]. It has been proposed that DENV NS3 interacts with fatty acid synthase (FASN) a critical lipid regulatory protein resulting in relocation of FASN to the site of viral replication, possibly modulating lipid composition in the virally induced membranes [3]. DENV NS3 is a multifunctional protein possessing both protease and RNA-stimulated NTPase and helicase activities [4], and the N-terminal protease activity is critically dependent upon the presence of a 40 amino acid cofactor present in the DENV NS2B protein [5]. We therefore sought to determine whether the NS2B-NS3 protease (NS2B-NS3pro) was capable of inducing changes in the cellular lipid profile, and subsequently whether co-expression of NS2B-NS3pro with a ZIKV VLP would improve VLP production. In addition catalytically inactive NS2B-NS3pro mutatnts were analysed in which a citical amino acid in the NS3pro catalytic triad was mutated, generating a catalytically inactive mutant [6, 7].

## Materials and methods

### Cells

HEK293T/17 cells (ATCC CRL-11268) were cultured at 37^o^C with 5% CO_2_ and maintained in DMEM (DMEM, GIBCO, Invitrogen, Grand Island, NY).

### Plasmid constructs

The ZIKV VLP construct used in this study was as previously described [8, 9]. The active ZIKV ZNS2B-NS3pro and inactive ZNS2B-NS3(S135A) were as previously described [10]. DENV NS2B-NS3pro and NS2B-NS3(S135A) were constructed in an essentially similar manner. The full cloning strategy can be found in supplemental materials.

### Construction of bicistronic NS3 protease and Zika VLPs expression plasmids

ZIKV VLP and NS3 protease (DENV2 NS2B-NS3pro (DWt), DENV2 NS2B-NS3(S135A) (DMut), ZIKV ZNS2B-NS3pro (ZWt) and ZIKV NS2B-NS3(S135A) (ZMut) and ZIKV NS3pro (ZNS3) were amplified from plasmid templates. The two proteins were joined together with a P2A cleavage site into pcDNA3.1+ (Thermo Fisher Scientific, Waltham, MA) expression plasmid [11]. Briefly, each sequence was amplified to contain half of the overlap P2A site using Phusion™ High-Fidelity DNA polymerase (Thermo Fisher Scientific) and the primers shown in Table S1. The two gene fragments for each construct were joined together by overlapping PCR with appropriate primers (Table S2) followed by restriction enzyme digestion, ligation and transformation. The bacteria containing plasmid were screened by size screening colony PCR. Positive clones were commercially sequenced (Macrogen, Seoul, Korea).

### Plasmid transfections and co-transfections

ZIKV and DENV NS2B-NS3pro and NS2B-NS3(S135A) plasmids were transfected into HEK293T/17 cells by the calcium precipitation method essentially as described previously [10] with some minor variations. Briefly, a day pre-transfection, HEK293T/17 cells were seeded (seed at 1 × 10^5^) into 24-well culture plates in DMEM supplemented with 10% heat-inactivated fetal bovine serum (FBS) without antibiotics at 37 ^o^C. A total of 1.0 µg of each plasmid was incubated with 2x HEPES buffered saline solution (HBS) and 2.5 M CaCl_2_ at room temperature for 30 min, before addition to cells and incubation under standard conditions. For co-transfections, 0.5 μg of each plasmid were mixed before incubation with 2x HEPES/2.5 M CaCl_2_ as for single plasmid transfections, before adding to 1.3 × 10^6^ HEK293T/17 cells grown in 6-well plates. For bicistronic constructs, 3.0 µg plasmid was incubated with 2x HEPES/2.5 M CaCl2 before being added to 1.3 × 10^6^ HEK293T/17 cells grown in 6-well plates. Although the amount of plasmids was different between transfection and co-transfection, expression levels were similar as shown by comparison of EGFP + ZIKV VLP and the bicistronic EGFP-ZIKV as assesses by fluorescent microscopy (Figure S1). For all VLP transfections at 24 h post transfection, culture media was replaced with Opti-MEM I Reduced Serum Media (Gibco, Thermo Fisher Scientific). Supernatant and cells were collected at 3 days post transfection and stored at -80^o^C until use. Experiments were performed as three independent biological replicates.

### Western blot analysis

To detect proteins in supernatants or cell lysates, 44 µl of supernatant was mixed with 11 µl of non-reducing lane marker sample marker (Thermo Fisher Scientific) while 20 µl of cell lysate was mixed with 5 µl reducing protein sample marker. Supernatants or lysates were then boiled for 5 min before loading 40 µl of supernatant or 20 µl of lysates onto 12% SDS-PAGE gels. After electrophoresis proteins were transferred to Amersham Protran 0.2 μm nitrocellulose membranes (GE Healthcare Life Science, Marlborough, MA). Membranes were blocked with 5% skim milk (Difco, Franklin Lakes, NJ) in 0.05% TBS-T and incubated with a specific primary antibody followed by appropriate secondary antibody conjugated HRP (Table S3). Chemiluminescence signal was developed using Immobilon Forte Western HRP substrate (Merck Millipore, Burlington, MA), and signal was collected using an AZURE c400 Gel Imaging System (Azure Biosystems, Inc., Dublin, CA) or X-ray film. Protein band intensity was analyzed using the Image J program version 1.47 [12].

### Electron microscopy of VLP preparations

Electron microscopy of VLPs produced from selected constructs was undertaken as described previously [1, 8].

### Statistical analysis

All statistical analyses of the numerical data were performed using Student T-test and one-way ANOVA on GraphPad Prism version 7.0.0 (GraphPad Software, CA, USA). The p values of less than 0.05 were considered statistically significant. Volcano plots analysis was undertaken using Agilent MassProfiler Professional software (version 10.0 Agilent Technologies, USA).

## Results

### FAME analysis of cells transfected with NS2B-NS3 pro constructs

**Fig. 1:**
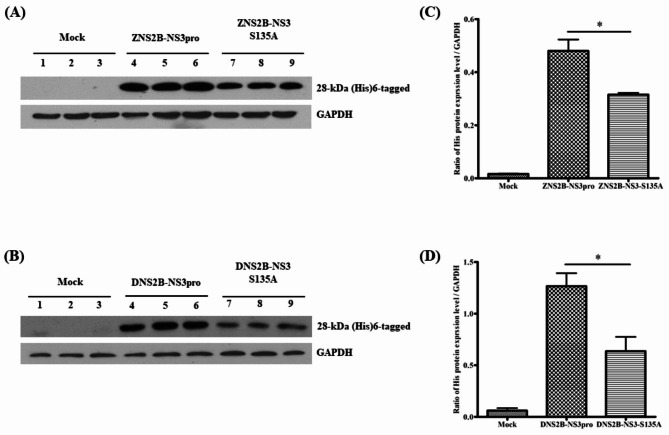
Expression of NS2B-NS3pro and NS2B-NS3pro (S135A) constructs in HEK293T/17 cells. (**A**) ZIKV NS2B-NS3pro and NS2B-NS3(S135A) and (**B**) DENV NS2B-NS3pro and NS2B-NS3(S135A) were transfected in HEK293T/17 cells. At day 2 post transfection cells were harvested and proteins extracted and separated by SDS-PAGE. Proteins were transferred to membranes which were probed successively with a mouse monoclonal anti-histidine-tag antibody and an anti-GAPDH antibody. (**C**, **D**) The expression of all NS2B-NS3 constructs was normalized to GAPDH. Error bars indicate the standard error of the mean from three independent biological replicates. P value * < 0.5. Bands shown are cropped from full length gels, and full length uncropped western blots are given in the supplemental materials

We have previously described [10] a ZIKV NS2B-NS3pro construct (ZNS2B-NS3pro), together with a catalytically inactive mutant (ZNS2B-NS3pro(S135A). We have additionally constructed an equivalent pair of plasmids based on DENV sequences, namely DNS2B-NS3pro and DNS2B-NS3pro(S135A). These constructs were separately transfected into HEK293T/17 cells. Expression of the constructs was confirmed by western blot analysis (Fig. 1). As observed previously [10] the inactive ZIKV construct (ZNS2B-NS3pro(S135A)) was expressed at a lower level than the active (ZNS2B-NS3pro) construct. Similarly, expression of the DENV constructs was also lower for the inactive construct (DNS2B-NS3pro(S135A)) than the active construct (DNS2B-NS3pro). However, the different expression levels were not considered to be critical in determining if these constructs altered the cellular lipid profile as analysis of the altered profiles was not directly quantitatively comparative.

To determine partial lipid profiles of the transfected cells, lipids were extracted, subjected to transesterification and the resultant fatty acid methyl esters (FAME) were analyzed by mass spectrometry. The data were annotated using Agilent MassProfiler Professional (MPP) software, and results were statistically analyzed. The resultant Volcano plots are shown in Figures S2 and S3, and Tables S4 to S7 show the statistically significant differently regulated FAME (as compared to mock) for DNS2B-NS3pro (Table S4), DNS2B-NS3pro(135 A) (Table S5), ZNS2B-NS3pro (Table S6) and ZNS2B-NS3pro(135 A) (Table S7). It was noted that the majority of fatty acid moieties were down-regulated, with only a few upregulated (Figures S2 and S3, Tables S4 to S7). Of particular note, both the inactive and active constructs for both DENV and ZIKV showed many of the same moieties being regulated in the same manner (bolded in the Table S4 to S7). This strongly suggests that NS2B-NS3pro can modulate lipid composition to at least some extent, irrespective of proteolytic activity. Collectively however, these results showed that both DENV and ZIKV constructs (active/inactive) were able to exert an effect on the cellular lipid profile.

To determine whether expression of NS2B-NS3pro (active or inactive) could enhance VLP expression, we undertook double transfections between a ZIKV VLP [8, 9] and the previously constructed ZIKV NS2B-NS3pro constructs [10], as well as the DENV NS2B-NS3pro constructs genrated in this study. In addition, as equal expression from two co-transfecting plasmids could not be ensured, we constructed vectors in which the two constructs (VLP and NS2B-NS3pro) were contained within the same expression vector, with a cell cleavable linker between the two constructs. All constructs consisted of the ZIKV VLP coupled with either EGFP and active and mutation inactive ZIKV and DENV NS2B-NS3pro, as well as two further constructs that contained just the ZIKV NS3 protease domain. A diagrammatic representation of the constructs is shown in Figure S4.

**Fig. 2:**
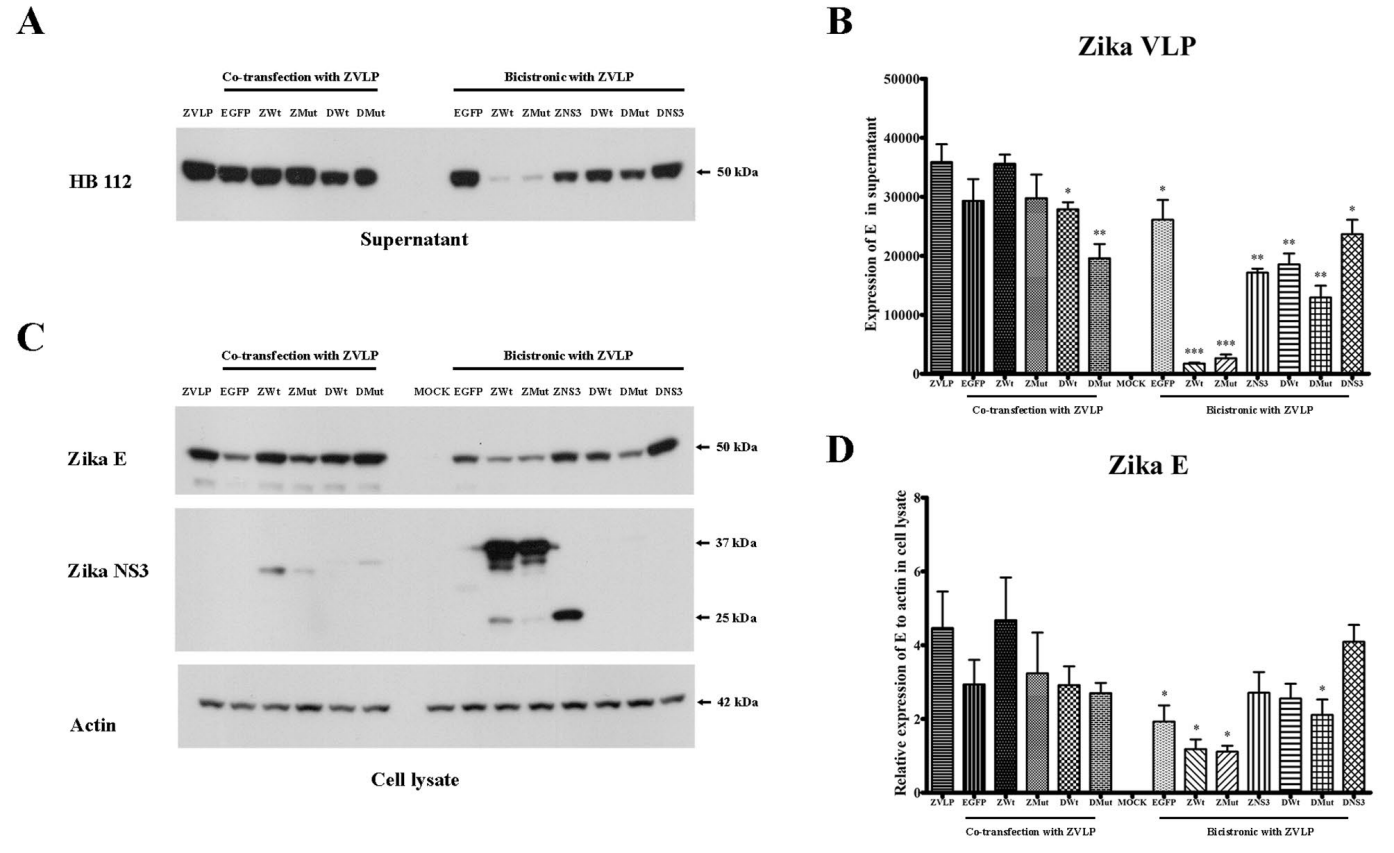
**Analysis of ZIKV E protein expression from bicistronic constructs.** HEK293T/17 cells were transfected with either a ZIKV VLP (ZVLP) or co-transfected with a ZIKV VLP and either a plasmid expressing EGFP, or a plasmid expressing ZIKV NS2B-NS3pro (Zwt), or a plasmid expressing ZIKV NS2B-NS3pro (S135A) (ZMut), or a plasmid expressing DENV NS2B-NS3pro (Dwt), or a plasmid expressing DENV NS2B-NS3pro (S135A) (DMut), or transfected with one of seven bicistronic vectors containing a ZIKV VLP downstream of either EGFP ((EGFP) or ZIKV NS2B-NS3pro (Zwt), ZIKV NS2B-NS3pro (S135A) (ZMut), ZIKV NS3pro (ZNS3), DENV NS2B-NS3pro (Dwt), DENV NS2B-NS3pro (S135A) (DMut) or DENV NS3pro (DNS3) separated by a cell cleavableP2A linker. At day 3 post transfection cells and supernatants were harvested and proteins extracted and separated by SDS-PAGE. (**A**) Proteins were transferred to membranes which were probed with a pan-flaviviral E protein antibody (HB112), or (**C**) a rabbit polyclonal anti-Zika virus E protein antibody (Zika **E**), or a rabbit polyclonal anti-Zika virus NS3 antibody (Zika NS3), or a mouse monoclonal anti-β actin antibody followed by appropriate HRP-conjugated secondary antibodies. Signals were captured by an AZURE C400 chemiluminescent gel imaging system and (**B**, **D**) quantitated using ImageJ. Error bars indicate the standard error of the mean from 3 independent biological replicates. Full length uncropped western blots are given in the supplemental materials. P value * < 0.5, ** < 0.01. Bands shown are cropped from full length gels, and full length uncropped western blots are given in the supplemental materials

All these constructs were transfected or co-tansfected (as appropriate) into HEK293T/17 cells in parallel with a single ZIKV VLP transfection, as well as a co-transfection between ZIKV VLP and a construct containing EGFP. After 3 days, the expression of E protein in the supernatant and cells was determined by western blot analysis (Fig. 2A, C) and the signal from three independent replicates was quantitated (Fig. 2B, D). In addition, the generation of VLPs from two of the bicistonic constructs was confirmed by electron microscopy (Figure S5). The results showed that co-transfection of any NS2B-NS3pro construct with the ZIKV VLP was either detrimental to VLP production, or had no significant effect on VLP levels (Fig. 2A, B). Co-transfection with EGFP had no statistically significant effect on VLP levels, albeit that levels were somewhat reduced (Fig. 2A, B). Although cellular E protein levels were reduced, the levels of the co-transfections were not significantly different from the levels seen with the single VLP transfection (Fig. 2C, D). Much greater detrimental effects were seen with the bicistronic constructs (Fig. 2). Expression of VLP was reduced for all constructs as compared to the single VLP transfection, sometimes markedly (e.g. ZIKV NS2B-NS3pro and ZIKV NS2B-NS3pro(S135A). The least affected (but still significantly reduced) bicistronic constructs were those that only contained the NS3pro domain (Fig. 2A, B). In contrast to the co-transfections, the bicistronic constructs showed significantly detrimental effects on cellular E protein expression, although again the NS3pro only constucts showed the least detrimental effects (Fig. 2C, D). We additionally investiagted the expression of NS3. We were unable to find an antibody that gave a signal for DENV NS3pro, as these are often raised against epitopes from the C-terminal of NS3, and the protease domain is N-terminal. However an anti-ZIKV NS3 antibody was able to detect ZIKV NS3pro (Fig. 2C) and quantitation showed that there while there was no significant difference in expression between ZIKV-NS2B-NS3pro and ZIKV NS2B-NS3pro (S135A), in either co-tansfection or in bicistronic expression, ZIKV NS3pro was expressed at a significantly lower level than the other two bicistronic constructs (Figure S6).

## Discussion

One of the hallmarks of *Flavivirus* infection is the remodeling of the cellular lipidome [2]. It is believed that this occurs to promote replication, both through increasing energy availability through increased autophagy [3, 13], as well as to change the lipid composition of cellular membranes to allow the necessary fluidity to promote the membrane curvature required to wrap membranes studded with E protein around the nucleocapsid during virion formation [14]. Based on previous studies [3] we hypothesized that the expression of the NS3 protease may have an impact on the cellular lipidome, and we were able to establish that the NS3 protease can modulate the lipid profile through a fatty acid methyl ester (FAME) analysis of cellular fatty acids after transfection with the active or inactive protease domain of both DENV and ZIKV. A number of fatty acids were regulated by both the active and inactive forms of the proteases, suggesting that while NS3 protease can indeed modulate the cellular lipid profile, proteolytic activity is not required. Previous studies have also shown that the expression of inactive NS2B-NS3 can exert cellular effects [15, 16]. The overall aim of this study was to investigate whether co-expression of NS2B-NS3pro was able to enhance VLP secretion. The results demonstrated that neither co-transfection nor co-expression via bicistronic vectors had any beneficial effects on VLP production, and at best effects were neutral and at worst positively detrimental. Why this result was obtained remains unknown. It is possible that the expression of (active/inactive) NS2B-NS3pro (alone) does not induce critical lipids required for greater membrane flexibility to enhance virion formation. It is also possible that the model that virion formation is enhanced by increased membrane flexibility is simply incorrect. Our study utilized expression of E protein in the supernatant as a marker for VLP production. However, the authors are unaware of any report in the literature that E protein is secreted from cells in a “free” form. Indeed, passage of nascent virions from the ER to the Golgi network generally requires recognition of the prM protein (and not the E protein) by KDEL receptors [17], while exocytosis of the fully mature virion involves recognition of E by the ESCRT machinery [18, 19]. The only free protein known to be secreted by infected cells is NS1. Thus it is reasonable to equate supernatant E protein with E protein in VLPs. While the concept of engineering cells to a more “infection like” status through co-expression of viral proteins that modulate the cellular lipidome remains sound, greater information on viral manipulation of the cellular lipidome is required before this can be achieved.

### Limitations

Viruses in the genus *Flavivirus* have a genome that encodes for three structural proteins and seven non-structural proteins, and any or all of these might be involved in manipulating the cellular lipidome. This study only looked at half of one of these proteins (NS3pro) together with its NS2B cofactor. In addition, the lipid analysis was confined to a FAME analysis, and so lipid categories other than fatty acyls were not investigated.

### Electronic supplementary material

Below is the link to the electronic supplementary material.

Supplementary Material 1

## Data Availability

Data is contained within the article, supplementary materials and data on the FAME analysis can be found in the publically accessible Mendeley Data, V1, doi: 10.17632/d7kjy4vmm6.1.

## Abbreviations

DENV: Dengue virus
EGFP: Enhanced green fluorescent protein
E: Envelope (protein)
FAME: Fatty acid methyl esters
NS: Non-structural (protein)
SDS-PAGE: Sodium dodecyl sulfate polyacrylamide gel electrophoresis
VLP: Virus like particles
ZIKV: Zika virus
