# Supplementary material for: Expression of dengue virus and Zika virus NS2B-NS3pro constructs alter cellular fatty acids, but co-expression with a Zika virus virus-like particle is detrimental to virus-like particle expression
Source: BMC Res Notes. 2023 Oct 27;16:296. doi: 10.1186/s13104-023-06572-z (PMC10605870; doi:10.1186/s13104-023-06572-z)
Supplement: Supplementary file 1 — Supplementary Material 1 [file 13104_2023_6572_MOESM1_ESM.pdf]

Supplemental materials

Expression of dengue virus and Zika virus NS2B-NS3pro constructs alter cellular fatty acids, but co-expression with a Zika virus virus-like particle is detrimental to virus-like particle expression

Suwipa Ramphan[1†], Nathamon Yimpring[1†], Chontida Tangsongcharoen[2], Suthatta Sornprasert[1], Atitaya Hitakarun[1], Wannapa Sornjai[1], Sittiruk Roytrakul[3], Atikorn Panya[4], Duncan R. Smith[1*]

[1]Institute of Molecular Biosciences, Mahidol University, Salaya, 73170, Thailand

(Su.R. r.suwipa.earn@gmail.com; N.Y jira.yim@gmail.com; S.S. suthatta.aum@gmail.com; A.H. tonar.scmi@gmail.com; W.S. wannapa.sor@mahidol.ac.th; D.R.S. duncan_r_smith@hotmail.com)

[2]Department of Medical Technology, Faculty of Allied Health Sciences, Burapha University, Chonburi 20130, Thailand (C.T. chontida.ta@go.buu.ac.th)

[3]Functional Proteomics Technology, National Center for Genetic Engineering and Biotechnology (BIOTECH), Thailand Science Park, Pathumthani 12120, Thailand (Si.R. sittiruk@biotec.or.th)

[4] Food Biotechnology Research Team, Functional Ingredients and Food Innovation Research Group, National Center for Genetic Engineering and Biotechnology (BIOTEC), Thailand Science Park, Pathumthani 12120, Thailand (A.P. atikorn.pan@biotec.or.th)

*****Correspondence: duncan_r_smith@hotmail.com

† These authors contributed equally.

Additional method

Plasmid constructs

DENV NS2B-NS3pro and NS2B-NS3(S135A) were constructed using cDNA of DENV 2 (strain 16681) as a template. The 48 amino acid central portion of the NS2B precursor protein (residues 49-96) and 185 amino acids of NS3 protease precursor protein (residues 1-185) were linked in frame with a Gly4SerGly4 linker (GGGGSGGGG) and cloned in frame with octahistidine (8xHis) tag at 3′ end of NS3 protease. The fragment of the NS2B cofactor linked with a Gly4SerGly4 was amplified with specific primers NheI-NS2B_Fw (5-CTAGCTAGCTAGATGGCCGATTTGGAACTGGAGAGA-3') and NS2B-GSG_Rev (5'-TCCTCCTCCTCCACTTCCTCCGCCTCCGGTCAGTGTTTG TTCTTCCTC) by polymerase chain reaction. A fragment of the NS3 protease linked with Gly4SerGly4 at 5' end and 8xHis tag at the 3' end was amplified with specific primers GSG-NS3_Fw (5'-GGAGGCGGAGGAAGTGGAGGAGGAGGAGCCGGAGTA TTGTGGGATGTTCCT-3') and NS3-His-XhoI_Rev (5'- CCGCTCGAGCGGTTAGTGGTGATGATGATGATGATGATG CTTTCGGAAAATGTCATCTTC). The PCR products of NS2B and NS3pro genes were assembled with primers NheI-NS2B_Fw and NS3-His-XhoI_Rev by overlapping fragment polymerase chain reaction. To generate the recombinant plasmid pcDNA3.1(+)_DENV2 NS2B-NS3pro, the assembled PCR products were cloned into NheI and XhoI sites of pcDNA3.1(+) vector (Thermo Fisher Scientific). The recombinant plasmid was transformed into *Escherichia coli* DH5α competent cells. Selected clones were screened by colony PCR and verified by commercial sequencing (Macrogen Inc., Korea).

To construct the mutant plasmid pcDNA3.1(+)_DENV2 NS2B-NS3(S135A) encoding DENV 2-NS2B cofactor domain linked with an enzymatically inactive NS3 protease by site-directed mutagenesis, the NS3 protease at the position of serine 135 (which is one of the conserved catalytic triad) was substituted with alanine in order to generate the inactive NS3 protease. The recombinant plasmid pcDNA3.1(+)_DENV2 NS2B-NS3pro was used as a template with site directed mutagenesis primers NS3-S135A_Fw (5'- TCCTGGAACGGCAGGATCTCCAATTATC-3') and NS3-S135A_Rev (5'-AGCTATTAACCTCTAGGACGGCAAGGTC-3') The PCR products were treated with DpnI and transformed into *E. coli* DH5α competent cells. Selected clones were screened by restriction endonuclease digestion and verified by acommercial sequencing (Macrogen Inc., Korea).

Lipid extraction

Lipids were extracted from cell pellets by a modified Bligh and Dyer protocol [Bligh and Dyer, 1959] using a ratio of Chloroform: Methanol: H_2O of 2: 2: 1. Briefly, 160 µl of chloroform (HPLC grade) was added to cells and then samples were incubated on ice for 1 h. After incubation, 320 µl of methanol (UHPLC grade) were added and tubes were agitated at 600 rpm at 15ºC for 30 min. After that, 150 µl of water (HPLC grade) was added and samples were further agitated at 600 rpm at 15ºC for 30 min. Finally, a further 160 µl of chloroform was added to the samples which were then agitated for 10 min before centrifugation at 13,500xg at 15ºC for 10 min. After centrifugation, the lower organic phase that contained of lipids was removed and transferred into a 3 ml furnace glass vial. The remaining aqueous phase was re-extracted once more with chloroform before centrifugation at 13,500xg at 15ºC for 10 min. The lower organic phase was removed and pooled

with the previous extraction. All lipid samples were dried under a gentle stream of nitrogen until the sample had evaporated.

Fatty acid profile using GC/Q-TOF

A total of 50 µl of myristic acid D27 (500 ppm in hexane) as an internal standard (IS) was added to the dried sample (from 100 µl of the lower organic phase at last step of the lipid extraction). The vial containing sample and IS was dried under vacuum at 60ºC for 30 min to remove the residual solvent. Subsequently 1% sulfuric acid in methanol (500 ul) was added to the vial, and the esterification reaction was initiated by incubation at 50 ºC for 2h. After that the vial was cooled to room temperature followed by the addition of 500 □l of 15% NaCl and 500 □l of hexane. The solution was mixed for 5 min using a multichannel mixer at 2400 rpm. After mixing, the samples were centrifuged at 3,000 x g for 5 min. The 200 □l-hexane supernatant layers were transferred into 1.5 ml V-shaped GC vials for GC analysis.

The samples were analyzed using a gas chromatography-quadrupole time of flight mass spectrometer (GC/Q-TOF, GC 7890B/MSD 7250, Agilent Technologies, USA) coupled to a PAL auto sampler system (CTC Analytics AG, Switzerland). An aliquot of the derivatized samples (1 µl) was injected into the GC/Q-TOF using a pulsed split mode with an injector temperature of 250°C, 45 psi until 0.5 min, a split ratio of 500 to 1, and a CP-Sil-88 column (100 m, 0.25 mm i.d., 0.20 µm film, Agilent Technologies, USA). Helium was used as the carrier gas with a constant flow rate of 1.2 ml/min. The GC oven was programmed as follows: The initial oven temperature was controlled at 100 °C, help for 0.5 min. Then, the temperature was ramped from 100 °C to 175 °C at the rate of 10 °C/min, and ramped from 175 °C to 185 °C at the rate of 5 °C/min, and

held for 5 min, finally ramped from 185 °C to 230 °C at the rate of 10 °C/min, and held for 20 min. Total run time was 39.5 min. The transfer line, ion source (EI), and quadrupole were set as 250 °C, 240 °C, and 150 °C, respectively. The mass spectrometer was operated in full scan mode, ranging from m/z 20-1200 with a data acquisition rate of 5 Hz. MS data was acquired using MassHunter software (version 10.0, Agilent Technologies, USA) utilizing three independent biological replicates to calculate the mean and the standard error.

The calibration curves were generated by mixing an equal volume of the mixture of fatty acid methyl esters (20 to 1,000 ppm) myristic acid-D27 methyl ester in hexane. Myristic acid-D27 methyl ester was prepared using the same method as described above; however, the concentration was increased twice in order to obtain the same concentration of the internal standard in the final mixture. Quantitative analyses were performed on Agilent MassHunter software (version 10.0 Agilent Technologies, USA), and exported into Microsoft Excel for further data processing.

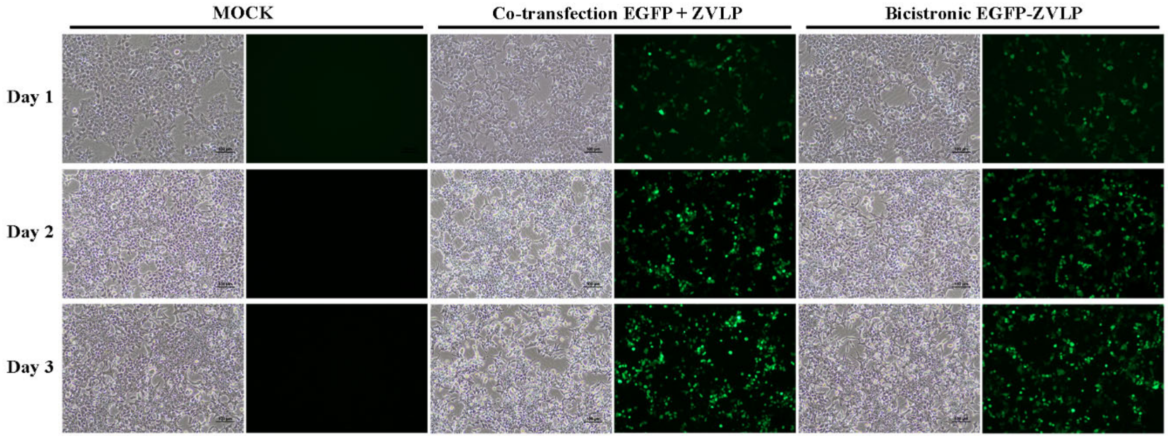

Supplemental Figure S1. Light and Fluorescent microscopy of transfected cells.

HEK293T/17 cells were co-transfected plasmids containing a ZIKV VLP and a plasmid expressing EGFP, or transfected with a bicistronic vector expressing EGFP and a ZIKV VLP separated by a cleavable linker. On day 3 post-transfection cells were examined under a fluorescent microscope.

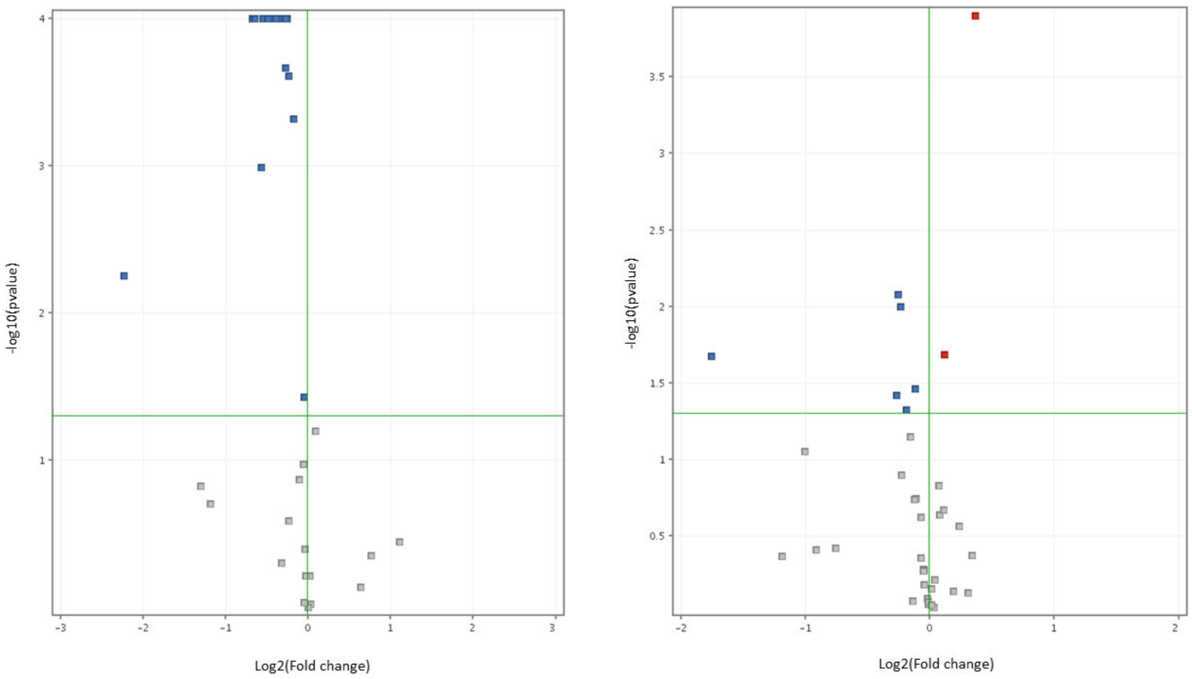

Supplemental Figure S2. DENV FAME Volcano plot.

Left: Data from DNS2B-NS3pro (a functional protease) transfected cells compared with mock transfected cells and Right: Data from DNS2B-NS3 (S135A) (a non-functional protease) transfected cells compared with mock transfected cells. Color by p-value fold change cut-offs: gray color represents failure to pass both cut-offs (not significant), blue color represents passing both cut-offs and is down regulated while red color represents the result passed both cut-offs and is up regulated.

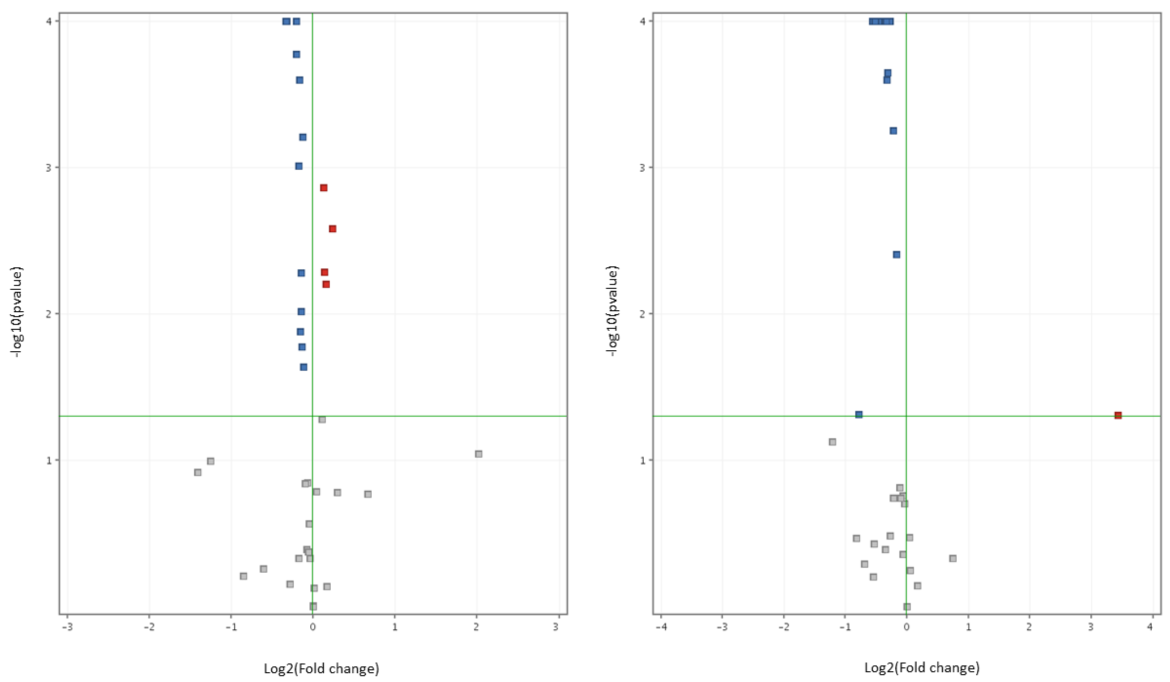

Supplemental Figure S3. ZIKV FAME Volcano plot.

Left: Data from ZNS2B-NS3pro (a functional protease) transfected cells compared with mock transfected cells and Right: Data from ZNS2B-NS3 (S135A) (a non-functional protease) transfected cells compared with mock transfected cells. Color by p-value fold change cut-offs: gray color represents failure to pass both cut-offs (not significant), blue color represents passing both cut-offs and is down regulated while red color represents the result passed both cut-offs and is up regulated.

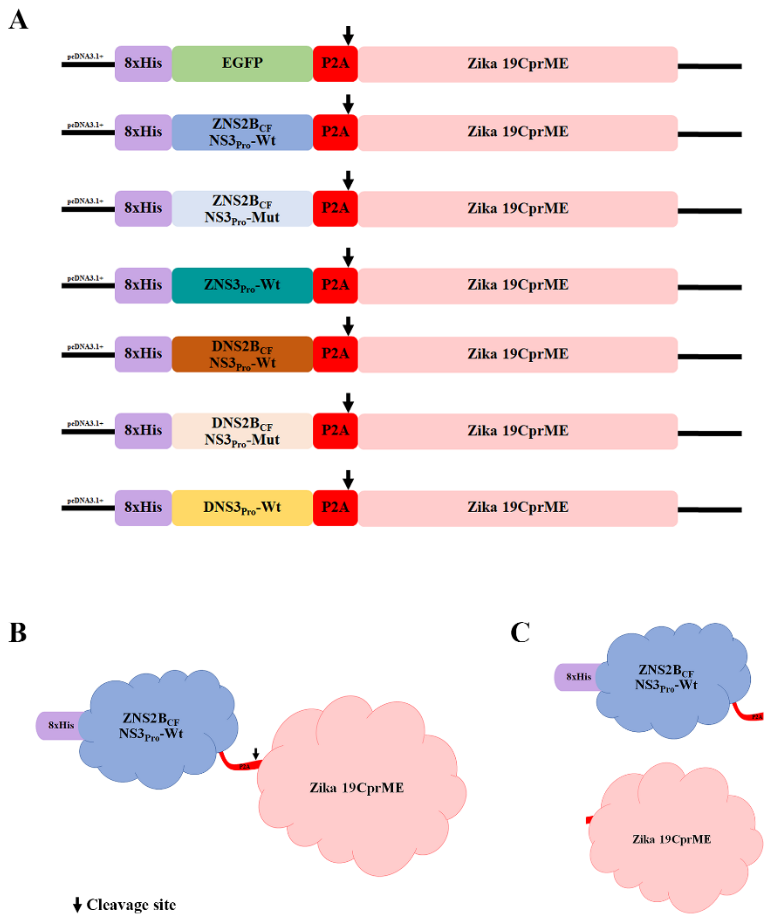

Supplemental Figure S4. Schematic of bicistronic expression plasmids.

Seven bicistronic expression vectors were constructed. Each construct contains the ZIKV VLP downstream of either EGFP or ZIKV NS2B-NS3pro, ZIKV NS2B-NS3pro (S135A), ZIKV NS3pro, DENV NS2B-NS3pro, DENV NS2B-NS3pro (S135A) or DENV NS3pro separated by a cell cleavable linker (P2A). The constructs are translated as a single polypeptide (B) which generates a (C) separate VLP after cell cleavage.

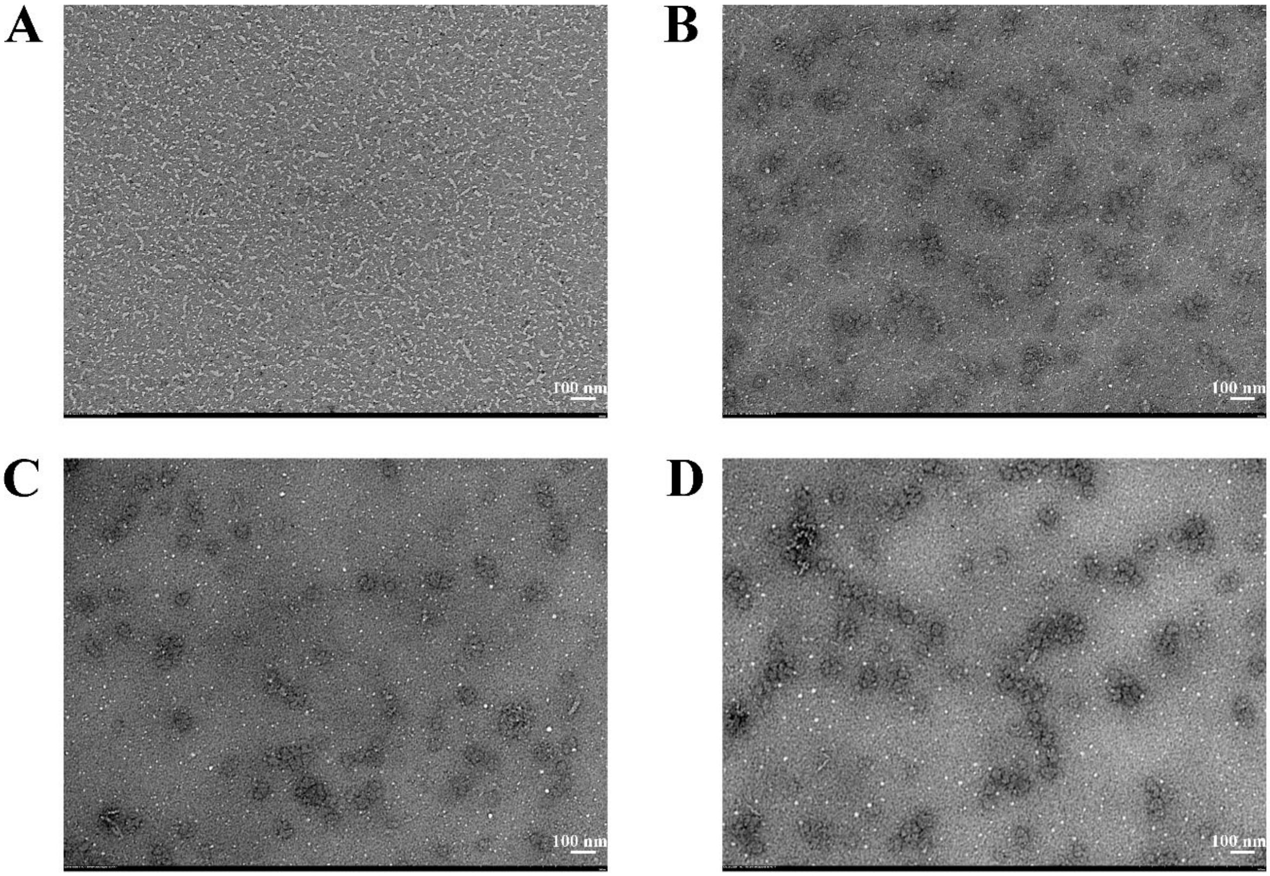

Supplemental Figure S5. ZIKV VLP analysis

HEK293T/17 cells were transfected with either (A) mock or a (B) ZIKV VLP (ZVLP) or a bicistronic plasmid vectors containing a ZIKV VLP downstream of either expressing (C) ZIKV NS3pro (ZNS3), or (D) DENV NS3pro (DNS3) that was cleaved to an ZNS3 or DNS3 and ZVLP. At 3 days post transfection the supernatant was collected, concentrated and purified by discontinuous sucrose gradient. ZIKV VLP particles were then observed by transmission electron microscopy (TEM, HT7700 Hitachi) with magnification 30,000X.

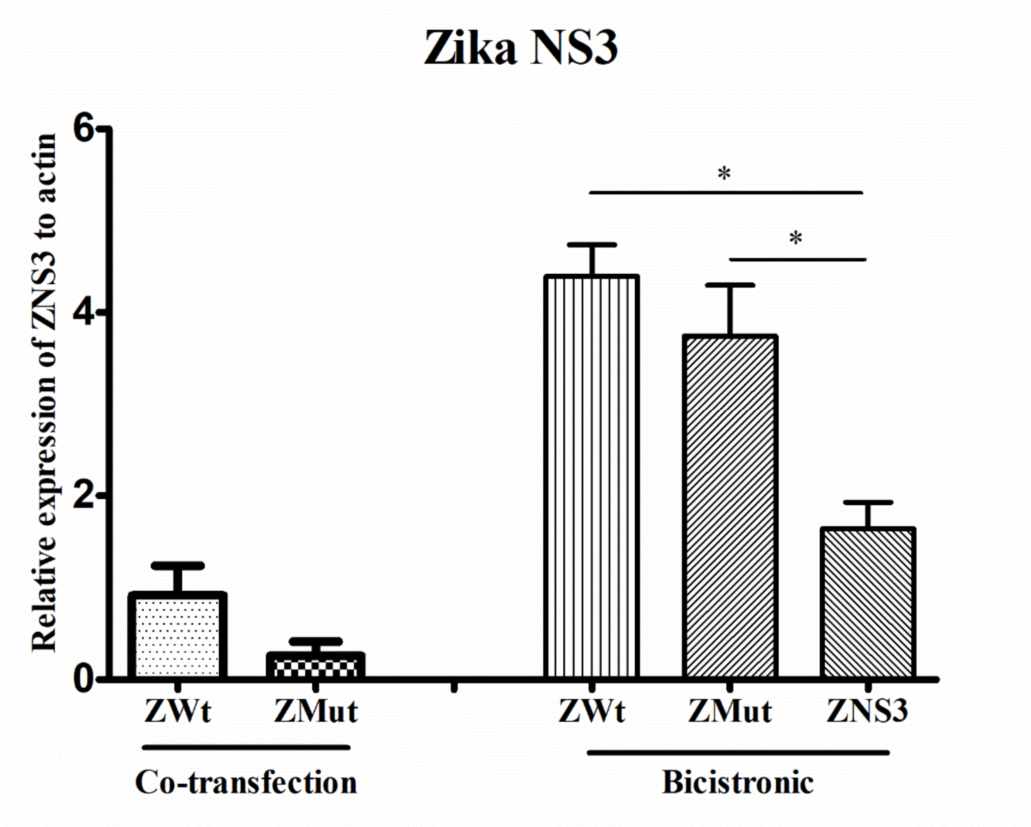

Supplemental Figure S6. Quantitation of ZIKV NS3 expression from co-transfection and bicistronic constructs.

Supplemental Table S1. Primers used to develop bicistronic vectors

Fragment	Primer F	Sequence
His-EGFP	8xHis-EGFP-NheI-F	TAGCTAGCGCCACCATGCATCATCATCATCATCACCACCACGGTGGTGTGAGCAAGGGCGAG
	EGFP-P2A-R	GTCTCCTGCTTGCTTTAACAGAGAGAAGTTCGTGGCTCCGGATCCCTTGTACAGCTCGTC
ZWt	8xHis-ZNS2B/3P2A-HindIII-F	TAAAGCTTGCCACCATGCATCATCATCATCATCACCACCACGGTGGTAGTGTGGACATGTAC
	ZNS2B/3-P2A-R	GTCTCCTGCTTGCTTTAACAGAGAGAAGTTCGTGGCTCCGGATCCCTGCTTCTTCTTCAGC
ZMut	8xHis-ZNS2B/3P2A-HindIII-F	TAAAGCTTGCCACCATGCATCATCATCATCATCACCACCACGGTGGTAGTGTGGACATGTAC
	ZNS2B/3-P2A-R	GTCTCCTGCTTGCTTTAACAGAGAGAAGTTCGTGGCTCCGGATCCCTGCTTCTTCTTCAGC
ZNS3pro	8XHis-ZNS3pro-P2A-F	TAAAGCTTGCCACCATGCATCATCATCATCATCACCACCACGGTGGTAGTGGTGCTCTATGGG
	ZNS2B/3-P2A-R	GTCTCCTGCTTGCTTTAACAGAGAGAAGTTCGTGGCTCCGGATCCCTGCTTCTTCTTCAGC
DWt	8xHis-DNS2B/3P2A-HindIII-F	TAAAGCTTGCCACCATGCATCATCATCATCATCACCACCACGGTGGTGCCGATTTGGAACTG
	DNS2B/3-P2A-R	GTCTCCTGCTTGCTTTAACAGAGAGAAGTTCGTGGCTCCGGATCCCTTTCGGAAAATGTCATC
DMut	8xHis-DNS2B/3P2A-HindIII-F	TAAAGCTTGCCACCATGCATCATCATCATCATCACCACCACGGTGGTGCCGATTTGGAACTG
	DNS2B/3-P2A-R	GTCTCCTGCTTGCTTTAACAGAGAGAAGTTCGTGGCTCCGGATCCCTTTCGGAAAATGTCATC
DNS3pro	8xHis-DNS3pro-P2A-F	TAAAGCTTGCCACCATGCATCATCATCATCATCACCACCACGGTGGTGCCGGAGTATTGTGGG
	DNS2B/3-P2A-R	GTCTCCTGCTTGCTTTAACAGAGAGAAGTTCGTGGCTCCGGATCCCTTTCGGAAAATGTCATC
ZVLP	P2A-19ZVLP-F	GCCACGAACTTCTCTCTGTTAAAGCAAGCAGGAGACGTGGAAGAAAACCCCGGTCCTAGAGGGACCGATAC
	P2A-19ZVLP-EcoRI-R	ATGAATTCTTATGCGGACACTG

Supplemental Table S2. Overlap PCR primers

PCR product	Primer F	Primer R
EGFP-ZVLP	8xHis-EGFP-NheI-F	P2A-19ZVLP-EcoRI-R
ZNS2B$_{CF}$-ZNS3$_{pro}$Wt-ZVLP	8xHis-ZNS2B/3P2A-HindIII-F	P2A-19ZVLP-EcoRI-R
ZNS2B$_{CF}$-ZNS3$_{pro}$Mut-ZVLP	8xHis-ZNS2B/3P2A-HindIII-F	P2A-19ZVLP-EcoRI-R
ZNS3$_{pro}$Wt-ZVLP	8XHis-ZNS3pro-P2A-F	P2A-19ZVLP-EcoRI-R
DNS2B$_{CF}$-DNS3$_{pro}$Wt-ZVLP	8xHis-DNS2B/3P2A-HindIII-F	P2A-19ZVLP-EcoRI-R
DNS2B$_{CF}$-DNS3$_{pro}$Mut-ZVLP	8xHis-DNS2B/3P2A-HindIII-F	P2A-19ZVLP-EcoRI-R
DNS3$_{pro}$Wt-ZVLP	8xHis-DNS3pro-P2A-F	P2A-19ZVLP-EcoRI-R

Supplemental Table S3. List of antibodies, dilutions and suppliers

Antibody	dilution	Catalog number	Company
Pan specific anti-flavivirus E protein (ATCC: HB-112)	1:500	[Henchal et al., 1982]	Antibody produced in house from hybridoma
Anti-His tag (AD1.1.20)	1:1,000	SC-53073	Santa Cruz Biotechnology, Inc., Dallas, TX
Anti-Zika virus Envelope protein	1:10,000	GTX133314	GeneTex, Irvine, CA
Anti-Zika virus NS3 protein	1:10,000	GTX133309	GeneTex, Irvine, CA
Anti-actin (I-19)	1:2,000	SC-1616	Santa Cruz Biotechnology, Inc., Dallas, TX
Anti-GAPDH	1:5,000	Sc-32233	Santa Cruz Biotechnology, Inc., Dallas, TX
Rabbit anti mouse-IgG HRP	1:8,000	AP160P	Merck Millipore
Goat anti rabbit-IgG HRP	1:8,000	31460	Thermo Fisher Scientific
Rabbit anti goat-IgG HRP	1:8,000	A5420	Sigma Aldrich, St. Louis, MO

Reference

Bligh EG, Dyer WJ. 1959. A rapid method of total lipid extraction and purification. Can J Biochem Physiol 37(8):911-917.

Henchal EA, Gentry MK, McCown JM, Brandt WE. 1982. Dengue virus-specific and flavivirus group determinants identified with monoclonal antibodies by indirect immunofluorescence. Am J Trop Med Hyg 31(4):830-836.

Supplemental Table S4. Differentially regulated FA compounds by active DENV protease. Fatty acid compounds differentially regulated by DNS2B-NS3pro as compared to mock. The result show down regulated compounds which passed both cut-offs (there were no upregulated compounds). Compounds in bold are common between active and inactive DENV NS2B-NS3pro constructs.

	**Compound**	**p ([Wild] Vs [Normal])**	**FC ([Wild] Vs [Normal])**	**Regulation ([Wild] Vs [Normal])**	**Log FC ([Wild] Vs [Normal])**
C6:0	**Hexanoic acid, methyl ester**	4.85E-04	1.128916	down	-0.17494
C8:0	Octanoic acid, methyl ester	0.037479427	1.034515	down	-0.04895
C14:0	Methyl tetradecanoate	1.62E-06	1.225951	down	-0.2939
C15:0	Pentadecanoic acid, methyl ester	3.33E-12	1.453542	down	-0.53957
C15:1 (cis-10)	**methyl cis 10-pentadecenoate**	2.31E-07	1.451635	down	-0.53768
C16:0	Hexadecanoic acid, methyl ester	1.22E-09	1.269236	down	-0.34396
C16:1 (cis-9)	9-Hexadecenoic acid, methyl ester, (Z)-	8.53E-08	1.315153	down	-0.39523
C17:0	**Heptadecanoic acid, methyl ester**	8.31E-11	1.458156	down	-0.54414
C18:0	Methyl stearate	1.69E-07	1.1962	down	-0.25846
C18:1 (trans-9)	9-Octadecenoic acid, methyl ester	0.001035235	1.484905	down	-0.57037
C18:1 (cis-9)	9-Octadecenoic acid (Z)-, methyl ester	4.17E-06	1.212381	down	-0.27784
C18:2 (all-cis-9,12)	**9,12-Octadecadienoic acid (Z,Z)-, methyl ester**	2.88E-09	1.601661	down	-0.67957
C20:0	Eicosanoic acid, methyl ester	1.74E-05	1.274066	down	-0.34944
C20:1 (cis-11)	cis-Methyl 11-eicosenoate	2.48E-04	1.177247	down	-0.23542
C22:0	Docosanoic acid, methyl ester	4.27E-06	1.29344	down	-0.37121
C20:3 (all-cis-8,11,14)	8,11,14-Eicosatrienoic acid methyl ester	9.75E-08	1.367415	down	-0.45145
C23:0	Tricosanoic acid, methyl ester	6.98E-09	1.403808	down	-0.48935
C20:4n6	5,8,11,14-Eicosatetraenoic acid, methyl ester, (all-Z)-	0.00561091	4.697778	down	-2.23198
C24:0	Tetracosanoic acid, methyl ester	2.19E-04	1.212935	down	-0.2785
C20:5 (all -cis-5,8,11,14,17)	**5,8,11,14,17-Eicosapentaenoic acid, methyl ester, (all-Z)-**	2.14E-09	1.562381	down	-0.64375
C22:6 (all-cis-4,7,10,13,16,19)	4,7,10,13,16,19-Docosahexaenoic acid methyl ester	4.99E-07	1.309117	down	-0.38859

Supplemental Table S5. FA compounds differentially regulated by inactive DENV protease. **F**atty acid compounds differentially regulated by DNS2B-NS3pro(135A) as compared to mock. The result show compounds down regulated and up regulated which passed both cut-offs. Compounds in bold are common between active and inactive DENV NS2B-NS3pro constructs.

	**Compound**	**p ([Mutant] Vs [Normal])**	**FC ([Mutant] Vs [Normal])**	**Regulation ([Mutant] Vs [Normal])**	**Log FC ([Mutant] Vs [Normal])**
C6:0	**Hexanoic acid, methyl ester**	0.03458	1.0861925	down	-0.11927979
C10:0	Decanoic acid, methyl ester	0.020691	1.0848289	up	0.117467456
C15:1 (cis-10)	**methyl cis 10-pentadecenoate**	0.038351	1.2052631	down	-0.26934814
C17:0	**Heptadecanoic acid, methyl ester**	0.047341	1.1393806	down	-0.1882497
C18:2 (all-cis-9,12)	**9,12-Octadecadienoic acid (Z,Z)-, methyl ester**	0.008385	1.1905951	down	-0.2516829
C22:1 (cis-13)	13-Docosenoic acid, methyl ester, (Z)-	1.25E-04	1.2872486	up	0.3642907
C22:2 (all-cis-13,16)	Methyl Z,Z 13,16-docosadienoate	0.021229	3.3740845	down	-1.7544961
C20:5 (all -cis-5,8,11,14,17)	**5,8,11,14,17-Eicosapentaenoic acid, methyl ester, (all-Z)-**	0.010085	1.1748745	down	-0.2325067

Supplemental Table S6. FA compounds differentially regulated active ZIKV protease. Fatty acid compounds differentially regulated active ZNS2B-NS3pro as compared to mock. The result show compounds down regulated and up regulated molecules which passed both cut-offs. Compounds in bold are common between active and inactive ZIKV NS2B-NS3pro constructs.

	**Compound**	**p ([Wild type] Vs [Normal])**	**Regulation ([Wild type] Vs [Normal])**	**FC ([Wild type] Vs [Normal])**	**Log FC ([Wild type] Vs [Normal])**
**C4:0**	Butanoic acid, methyl ester	0.005200198	up	1.098049	0.134942
**C6:0**	**Hexanoic acid, methyl ester**	0.016890224	down	-1.1007	-0.13842
**C10:0**	Decanoic acid, methyl ester	0.001387321	up	1.09351	0.128966
**C15:0**	**Pentadecanoic acid, methyl ester**	2.53E-04	down	-1.12077	-0.16449
**C16:0**	**Hexadecanoic acid, methyl ester**	4.92E-07	down	-1.14943	-0.20092
**C16:1 (cis-9)**	**9-Hexadecenoic acid, methyl ester, (Z)-**	0.005264493	down	-1.11034	-0.151
**C17:0**	**Heptadecanoic acid, methyl ester**	1.68E-04	down	-1.15544	-0.20844
**C18:0**	Methyl stearate	6.24E-04	down	-1.0929	-0.12816
**C18:2 (all-cis-9,12)**	**9,12-Octadecadienoic acid (Z,Z)-, methyl ester**	2.06E-06	down	-1.25032	-0.3223
**C20:0**	**Eicosanoic acid, methyl ester**	0.013247293	down	-1.11466	-0.1566
**C20:1 (cis-11)**	cis-Methyl 11-eicosenoate	0.006279776	up	1.11145	0.152444
**C20:3 (all-cis-8,11,14)**	**8,11,14-Eicosatrienoic acid methyl ester**	0.009617892	down	-1.10796	-0.1479
**C22:1 (cis-13)**	13-Docosenoic acid, methyl ester, (Z)-	0.002615343	up	1.178098	0.23646
**C23:0**	**Tricosanoic acid, methyl ester**	9.81E-04	down	-1.13342	-0.18068
**C20:5 (all -cis-5,8,11,14,17)**	**5,8,11,14,17-Eicosapentaenoic acid, methyl ester, (all-Z)-**	1.86E-06	down	-1.26221	-0.33595
**C22:6 (all-cis-4,7,10,13,16,19)**	**4,7,10,13,16,19-Docosahexaenoic acid methyl ester**	0.023272282	down	-1.08507	-0.11779

Supplemental Table S7. FA compounds differentially regulated by inactive ZIKV protease. Fatty acid compounds differentially regulated byZNS2B-NS3 (135A) as compared to mock. The result show compounds down regulated and up regulated which passed both cut-offs. Compounds in bold are common between active and inactive ZIKV NS2B-NS3pro constructs.

	**Compound**	**p ([Mutant] Vs [Normal])**	**Regulation ([Mutant] Vs [Normal])**	**FC ([Mutant] Vs [Normal])**	**Log FC ([Mutant] Vs [Normal])**
**C6:0**	**Hexanoic acid, methyl ester**	5.58E-04	down	-1.16898	-0.22525
**C14:0**	Methyl tetradecanoate	1.49E-05	down	-1.2199	-0.28676
**C14:1**	Methyl myristoleate	0.049424	up	10.78521	3.430983
**C15:0**	**Pentadecanoic acid, methyl ester**	5.04E-07	down	-1.29238	-0.37003
**C15:1 (cis-10)**	methyl cis 10-pentadecenoate	1.23E-06	down	-1.39409	-0.47933
**C16:0**	**Hexadecanoic acid, methyl ester**	3.01E-09	down	-1.27402	-0.34939
**C16:1 (cis-9)**	**9-Hexadecenoic acid, methyl ester, (Z)-**	4.40E-05	down	-1.20736	-0.27185
**C17:0**	Heptadecanoic acid, methyl ester	3.37E-07	down	-1.31492	-0.39498
**C18:0**	**Methyl stearate**	1.93E-07	down	-1.22228	-0.28957
**C18:1 (cis-9)**	9-Octadecenoic acid (Z)-, methyl ester	0.003937	down	-1.12785	-0.17358
**C18:2 (all-cis-9,12)**	**9,12-Octadecadienoic acid (Z,Z)-, methyl ester**	1.63E-06	down	-1.48066	-0.56624
**C20:0**	**Eicosanoic acid, methyl ester**	1.30E-05	down	-1.33247	-0.4141
**C22:0**	Docosanoic acid, methyl ester	2.26E-04	down	-1.23948	-0.30973
**C20:3 (all-cis-8,11,14)**	**8,11,14-Eicosatrienoic acid methyl ester**	2.84E-06	down	-1.27923	-0.35528
**C23:0**	Tricosanoic acid, methyl ester	3.76E-06	down	-1.28682	-0.36381
**C20:4n6**	5,8,11,14-Eicosatetraenoic acid, methyl ester, (all-Z)-	0.048714	down	-1.72132	-0.78351
**C24:0**	**Tetracosanoic acid, methyl ester**	2.54E-04	down	-1.25193	-0.32415
**C20:5 (all -cis-5,8,11,14,17)**	**5,8,11,14,17-Eicosapentaenoic acid, methyl ester, (all-Z)-**	1.76E-07	down	-1.43299	-0.51903
**C22:6 (all-cis-4,7,10,13,16,19)**	**4,7,10,13,16,19-Docosahexaenoic acid methyl ester**	9.13E-05	down	-1.26942	-0.34417

Full length western blots

File name: ZIKA_ (Mock_pro_S135A)

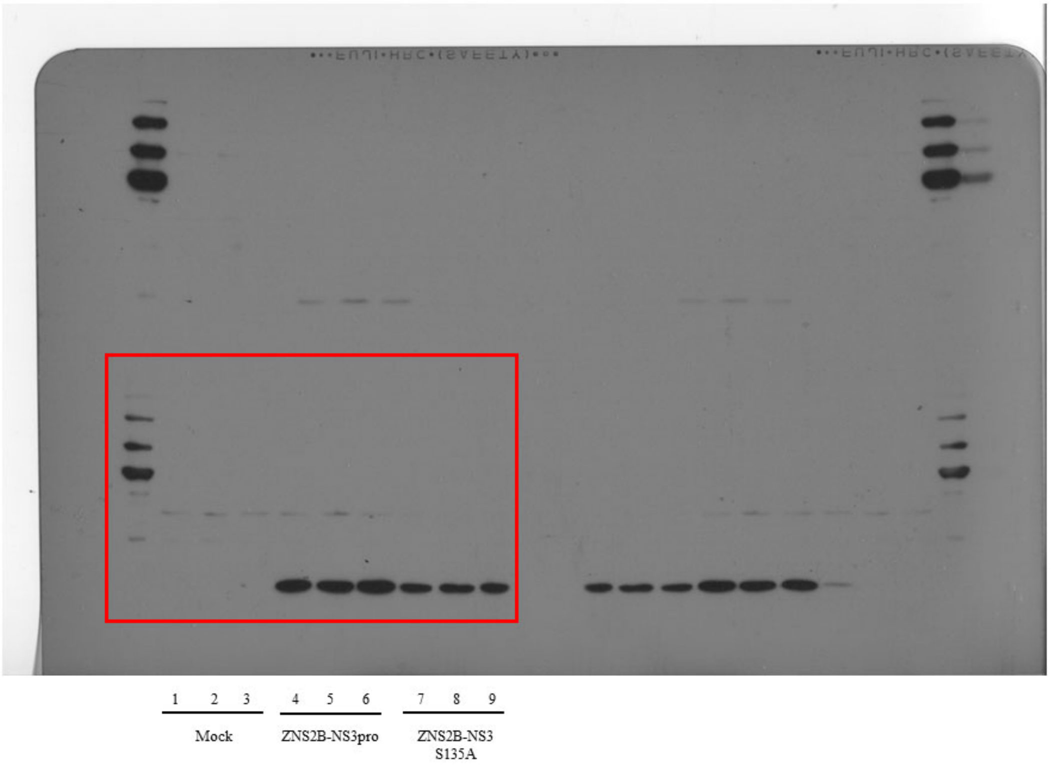

Figure 1A

File name: ZIKA_**GAPDH**

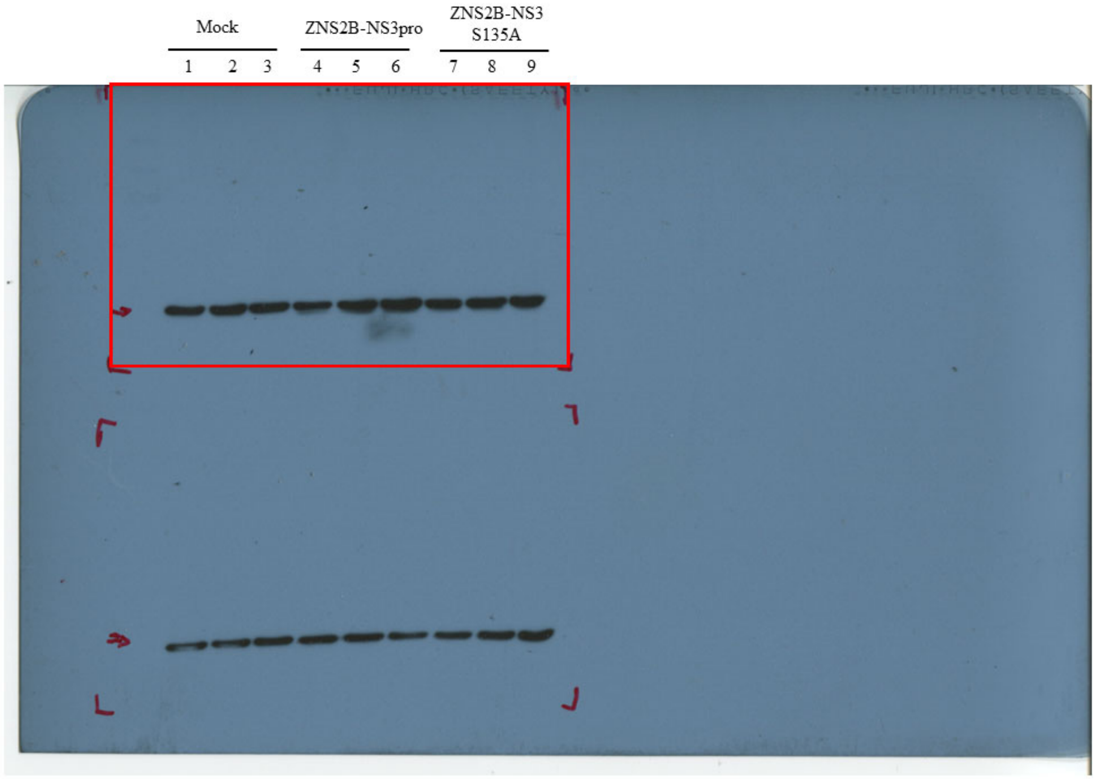

Figure 1A

File name: DENV _ (Mock_pro_S135A)

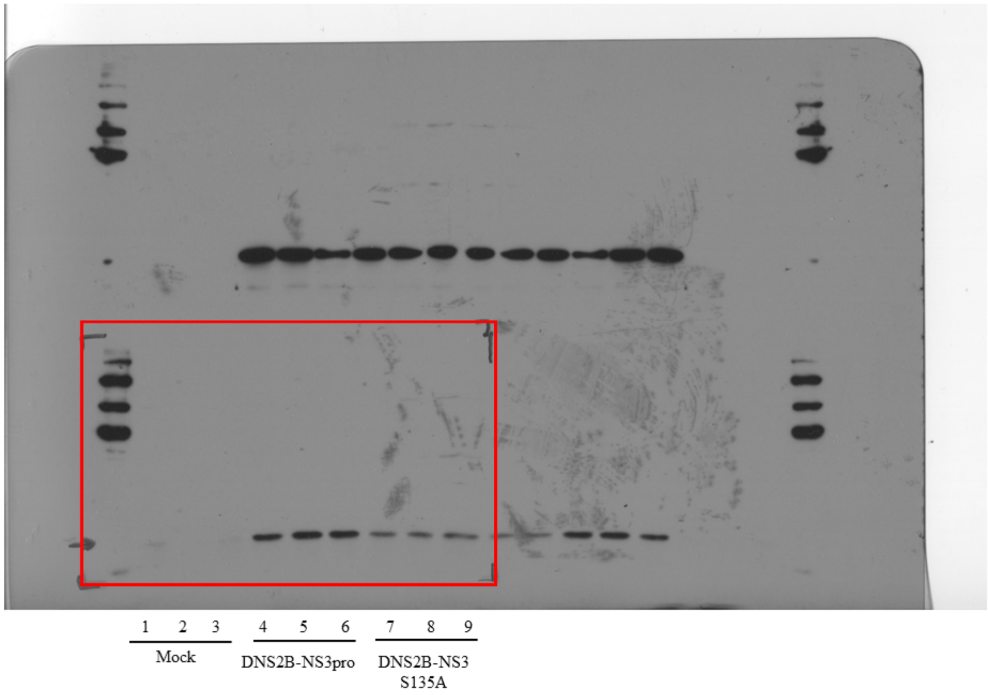

Figure 1B

File name: DENV_**GAPDH**

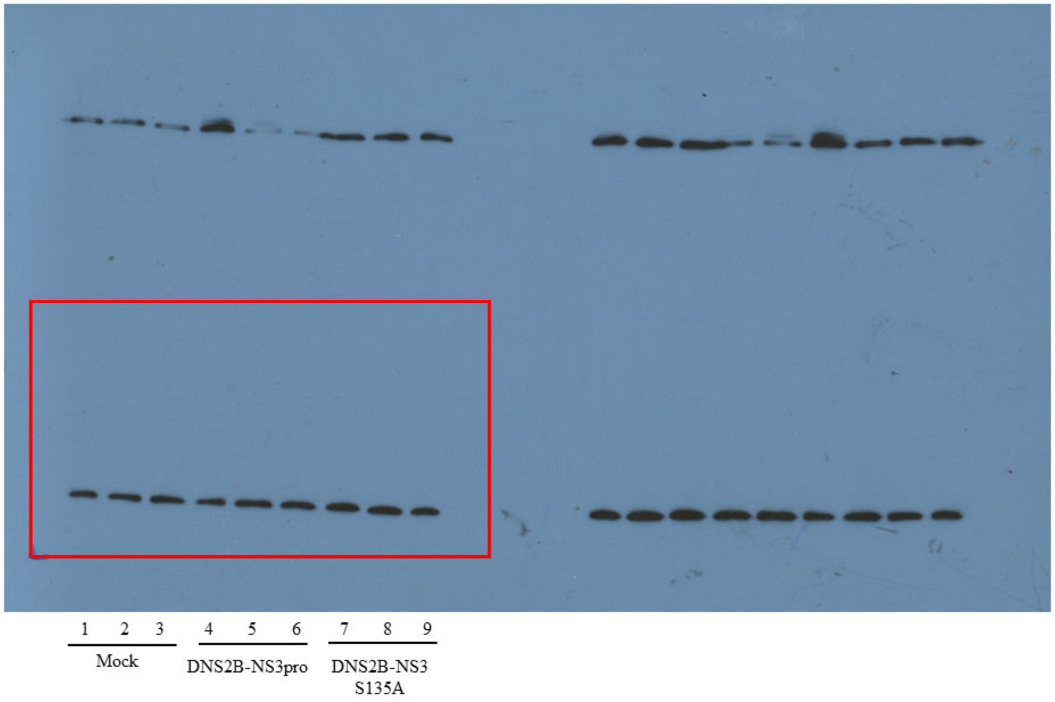

Figure 1B

HB112

Replicate 1

Replicate 2

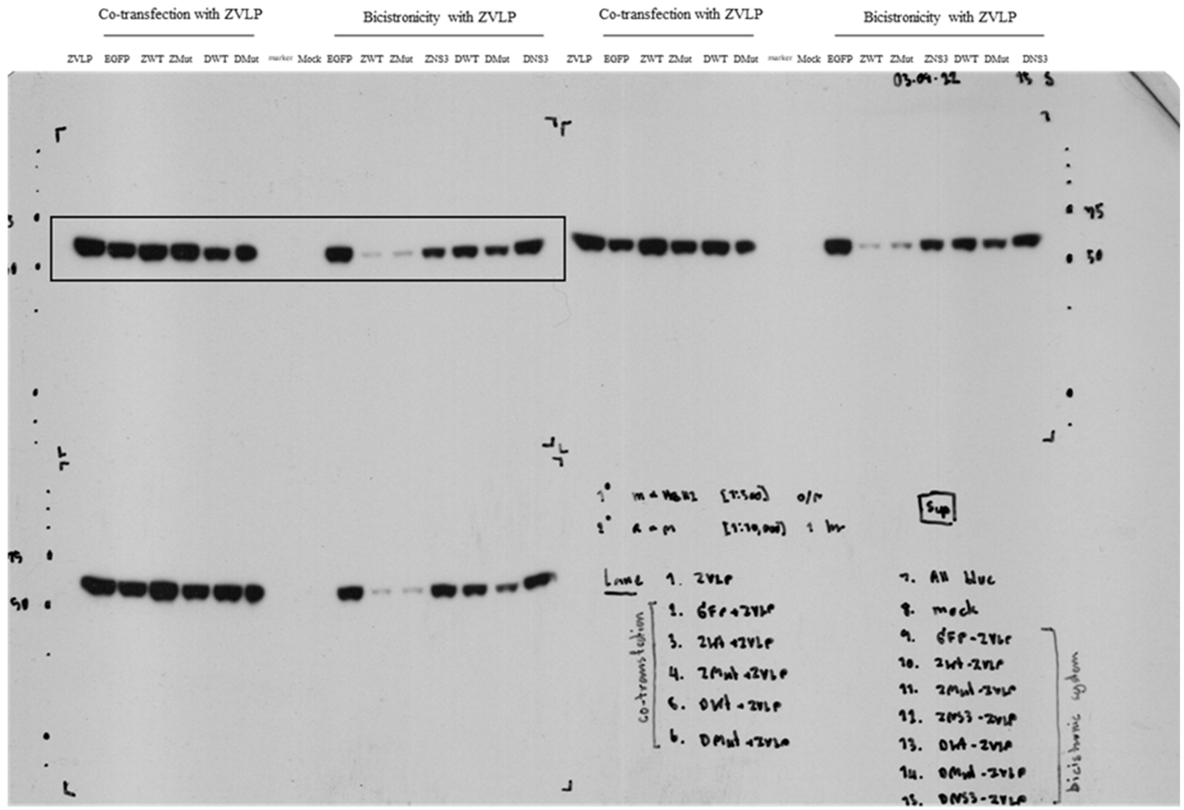

Replicate 3

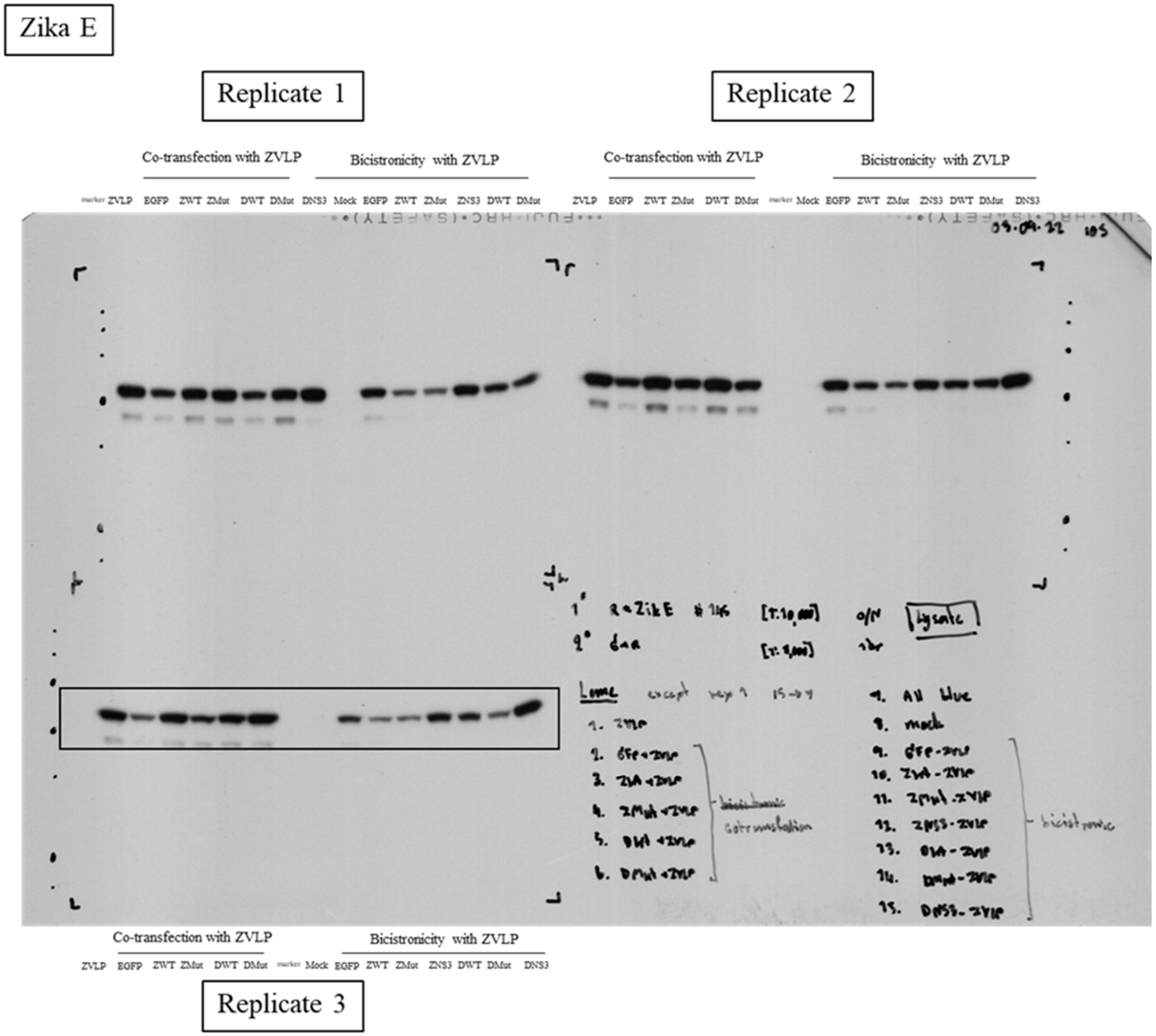
Zika E
Replicate 1
Replicate 2
Co-transfection with ZVLP
Bicistronicity with ZVLP
Co-transfection with ZVLP
Bicistronicity with ZVLP
marker ZVLP EGFP ZWT ZMut DWT DMut DNS3 Mock EGFP ZWT ZMut ZNS3 DWT DMut
ZVLP EGFP ZWT ZMut DWT DMut marker Mock EGFP ZWT ZMut ZNS3 DWT DMut DNS3
Co-transfection with ZVLP
Bicistronicity with ZVLP
ZVLP EGFP ZWT ZMut DWT DMut marker Mock EGFP ZWT ZMut ZNS3 DWT DMut DNS3
Replicate 3

Zika NS3

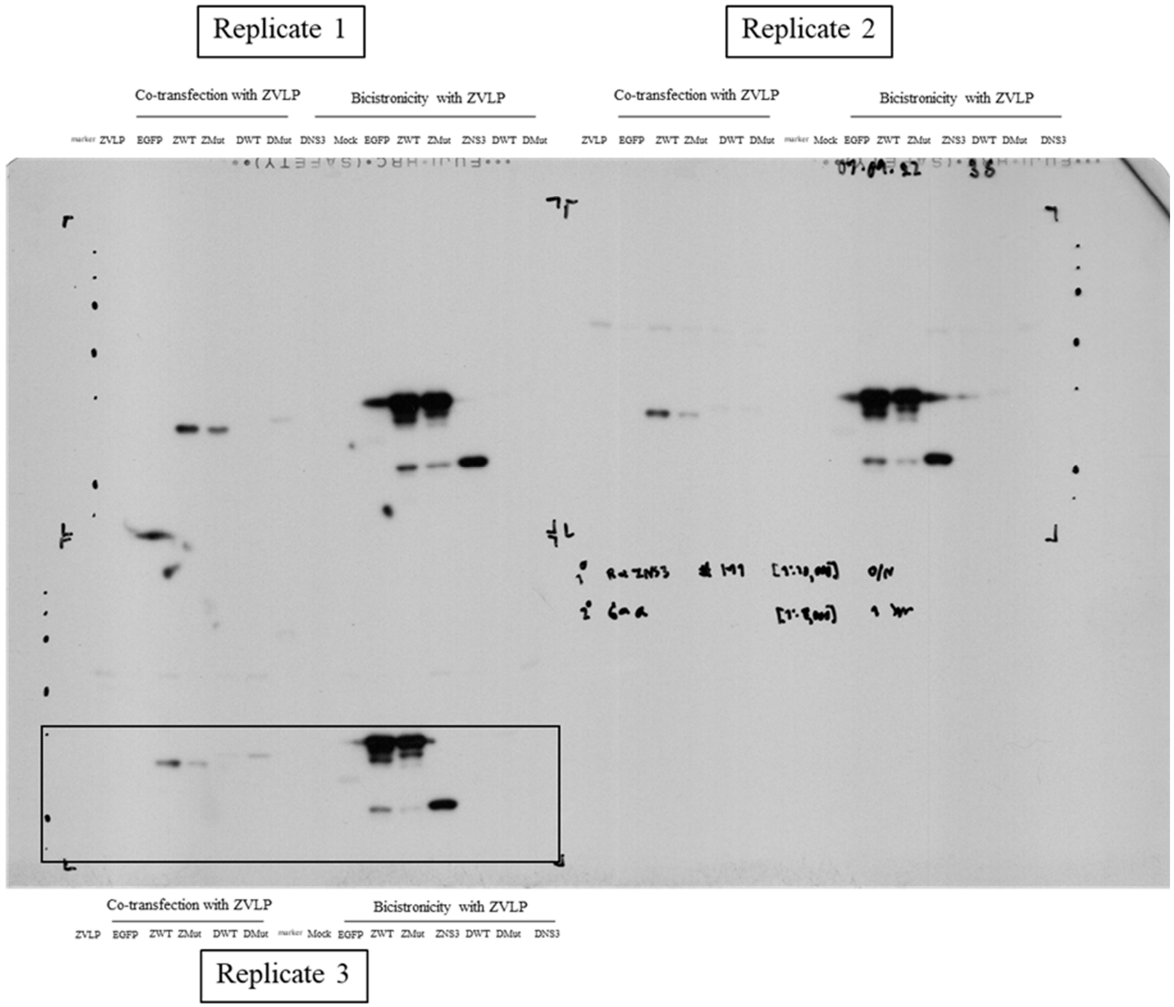
Replicate 1
Co-transfection with ZVLP
Bicistronicity with ZVLP
marker ZVLP EGFP ZWT ZMut DWT DMut DNS3 Mock EGFP ZWT ZMut ZNS3 DWT DMut
Replicate 2
Co-transfection with ZVLP
Bicistronicity with ZVLP
ZVLP EGFP ZWT ZMut DWT DMut marker Mock EGFP ZWT ZMut ZNS3 DWT DMut DNS3
Co-transfection with ZVLP
Bicistronicity with ZVLP
ZVLP EGFP ZWT ZMut DWT DMut marker Mock EGFP ZWT ZMut ZNS3 DWT DMut DNS3
Replicate 3

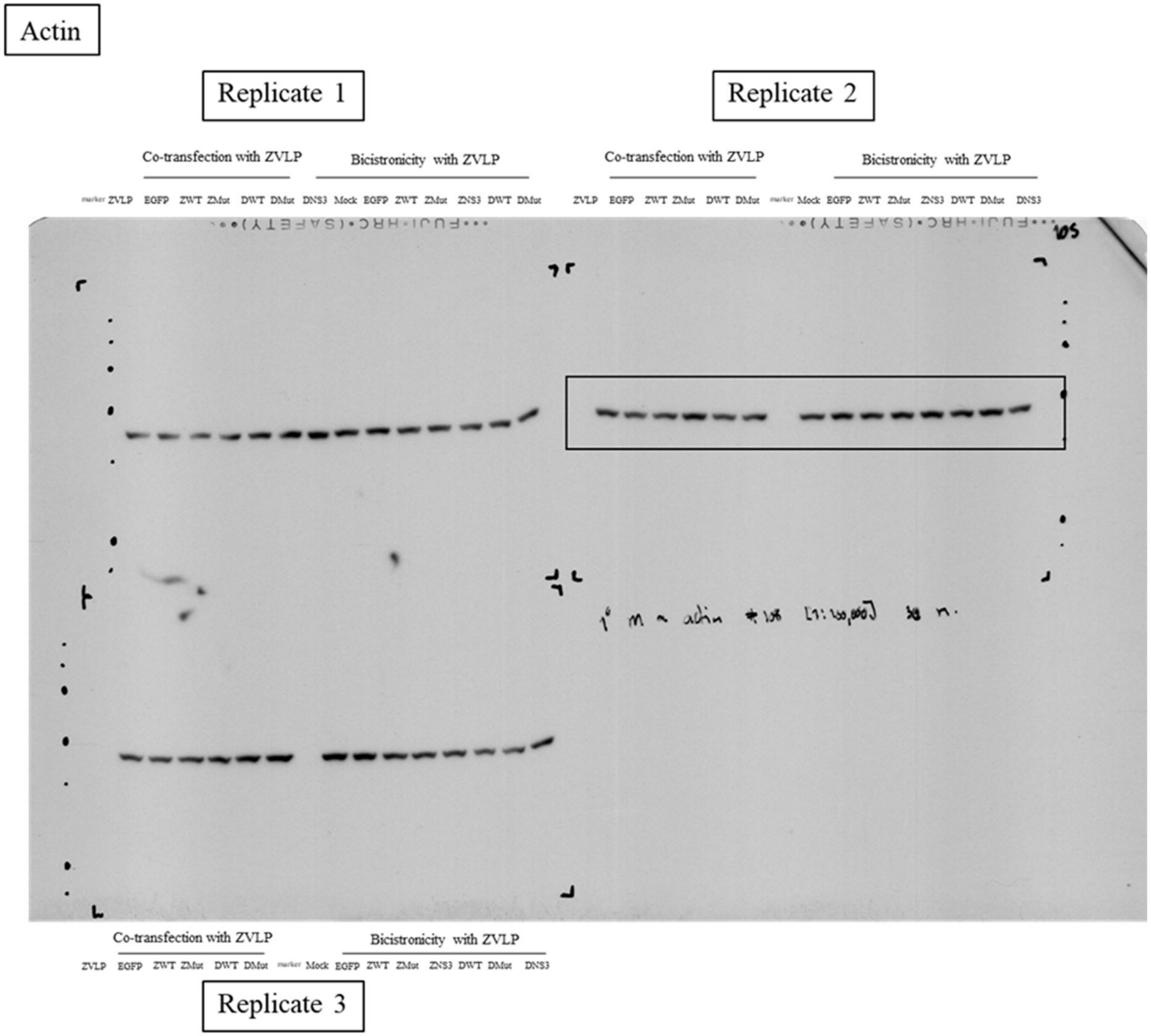
Actin
Replicate 1
Co-transfection with ZVLP
Bicistronicity with ZVLP
marker ZVLP EGFP ZWT ZMut DWT DMut DNS3 Mock EGFP ZWT ZMut ZNS3 DWT DMut
Replicate 2
Co-transfection with ZVLP
Bicistronicity with ZVLP
ZVLP EGFP ZWT ZMut DWT DMut marker Mock EGFP ZWT ZMut ZNS3 DWT DMut DNS3
Co-transfection with ZVLP
Bicistronicity with ZVLP
ZVLP EGFP ZWT ZMut DWT DMut marker Mock EGFP ZWT ZMut ZNS3 DWT DMut DNS3
Replicate 3
